# Migrated Sacral Facet Joint Cyst Mimicking a Tarlov Cyst: A Case Report

**DOI:** 10.1155/crnm/2679509

**Published:** 2026-04-24

**Authors:** Evangelos A. Christodoulou, Konstantinos Kafchitsas

**Affiliations:** ^1^ St. Vinzenz Hospital, Spine & Pain Clinic, Schloßstrasse 85, Düsseldorf, 40477, Germany; ^2^ Department of Orthopaedic Surgery, Asklepios Hospital Lindenlohe 18, Schwandorf, 92421, Germany

**Keywords:** differential diagnosis, facet joint cyst, lumbar spine, MRI, radicular pain, Tarlov cyst

## Abstract

We report the case of a 78‐year‐old male presenting with debilitating left‐sided S1 radicular pain, associated with a positive Lasègue sign and numbness in the S1 dermatome. The patient exhibited mild motor deficits, but his pain was severe enough to impair standing and ambulation. Magnetic resonance imaging (MRI) revealed a cystic lesion at the S1 level; however, the exact etiology remained unclear—differential diagnoses included a Tarlov cyst or a facet joint cyst. No other spinal comorbidities were noticed. Initial conservative management, including intravenous analgesics and C‐arm–guided S1 nerve root blocks, failed to provide relief. Due to persistent symptoms, surgical exploration was undertaken. Intraoperatively, a facet joint cyst was identified and successfully excised. Postoperative recovery was uneventful, and the patient reported significant pain relief and functional improvement. This case highlights an unusual presentation of a facet joint cyst located at the sacral level without a visible connection to the facet joint, raising diagnostic challenges on imaging.

## 1. Introduction

Facet joint cysts (FJCs) are common fluid‐filled lesions that arise from the facet joints of the spine, with a prevalence of up to 6.5%. They are often seen in the lumbar region and are associated with degenerative changes in the facet joints. Although almost half of the FJCs are asymptomatic (46%), when they cause nerve root compression, they can result in significant symptoms, such as radiculopathy, pain, and neurological deficits [1–3]. Tarlov cysts (TCs), or perineural cysts, are cystic dilatations of the spinal nerve root sheath that can occur at any spinal level. They are most commonly identified in the sacral region but have also been reported in the lumbar, thoracic, and cervical spine [4]. The vast majority are incidental radiological findings and asymptomatic [5]. The global pooled prevalence of TCs was found to be 4.18% [6]. Given the proximity of both lesions to the nerve roots, they can present with similar symptoms, often leading to diagnostic challenges.

This case presents a lumbar FJC that mimicked a TC, focusing on the radiological and clinical features that distinguish these two entities. The case highlights the importance of differentiating FJCs from TCs to ensure proper diagnosis and treatment.

## 2. Case Presentation

A 78‐year‐old male patient presented with a 1‐week history of worsening low back pain radiating down the left leg. He described the pain as sharp and shooting, accompanied by numbness in the left foot. The patient also reported intermittent episodes of weakness in the left lower limb, particularly during plantarflexion. No bowel or bladder dysfunction was noted.

On physical examination, the patient was alert and oriented. Neurological assessment revealed decreased sensation in the left S1 dermatome. Muscle strength testing demonstrated mild weakness in left plantarflexion (4/5). The straight leg raise test was positive at 30° on the left, indicating nerve root irritation. Deep tendon reflexes were normal, and there were no signs of myelopathy.

**FIGURE 3:**
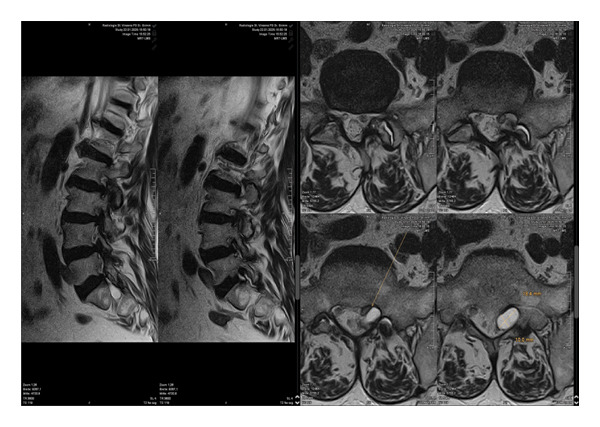
The cyst shows no well‐defined attachment to the facet joint, which indicates a facet joint cyst. However, further evaluation of the imaging revealed synovial fluid tracking along the joint capsule in the left L5–S1 facet joint.

Magnetic resonance imaging of the lumbar spine revealed a well‐circumscribed cystic lesion measuring 1.84 × 1 cm, located dorsolaterally on the left side of the sacral spinal canal, inferior to the left S1 pedicle, causing compression of the exiting left S1 nerve root (Figures 1(a), 1(b), and 1(c)). The lesion was fluid‐filled and demonstrated signal characteristics similar to cerebrospinal fluid (CSF; T2 hyperintense and T1 hypointense), thereby radiologically resembling a TC (Figures 2(a), 2(b), and 2(c)). No definite or direct attachment or communication with the adjacent facet joint was identified on imaging, and the lesion did not appear to arise from or incorporate the nerve root sheath, as is typically seen in true Tarlov (perineural) cysts. However, degenerative changes of the left L5–S1 facet joint were noted, with synovial fluid tracking along the joint capsule. Although these findings did not demonstrate a clear anatomical connection to the cyst, they raised suspicion for a synovial (facet joint) cyst and therefore led to its inclusion in the differential diagnosis (Figure 3).

FIGURE 1Sagittal (a) and axial (b and c) T2‐weighted MRI images showing a cystic lesion inferior to the left S1 pedicle causing compression to the S1 exiting left nerve root (arrow).

**Figure:**
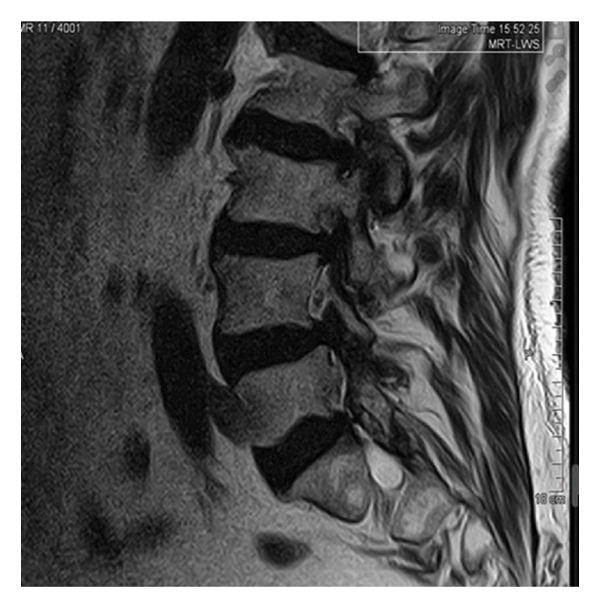
(a)

**Figure:**
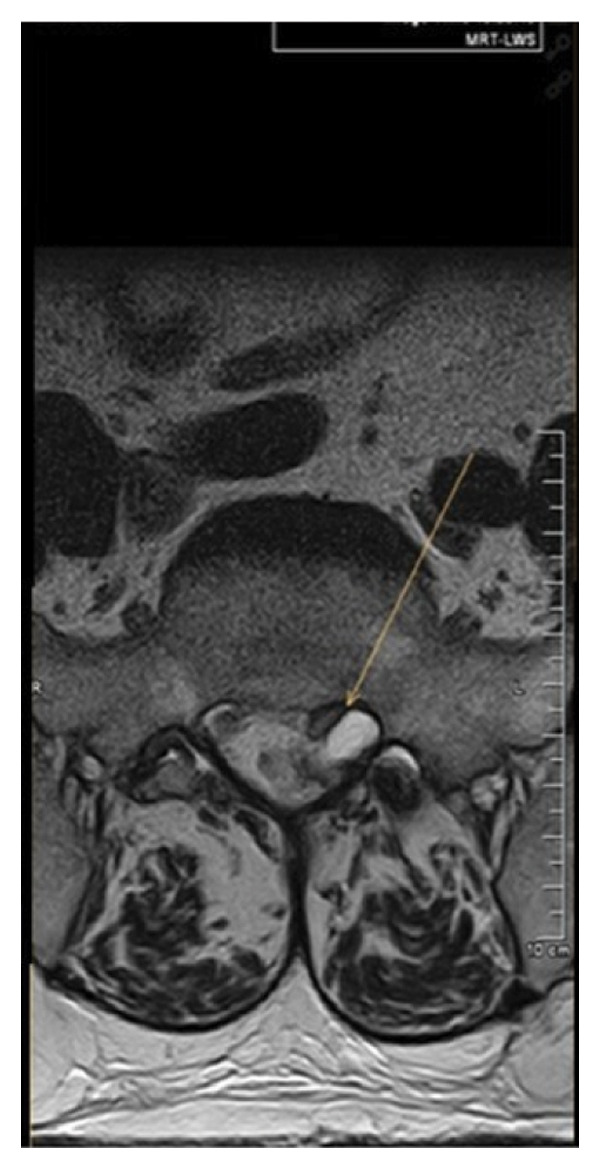
(b)

**Figure:**
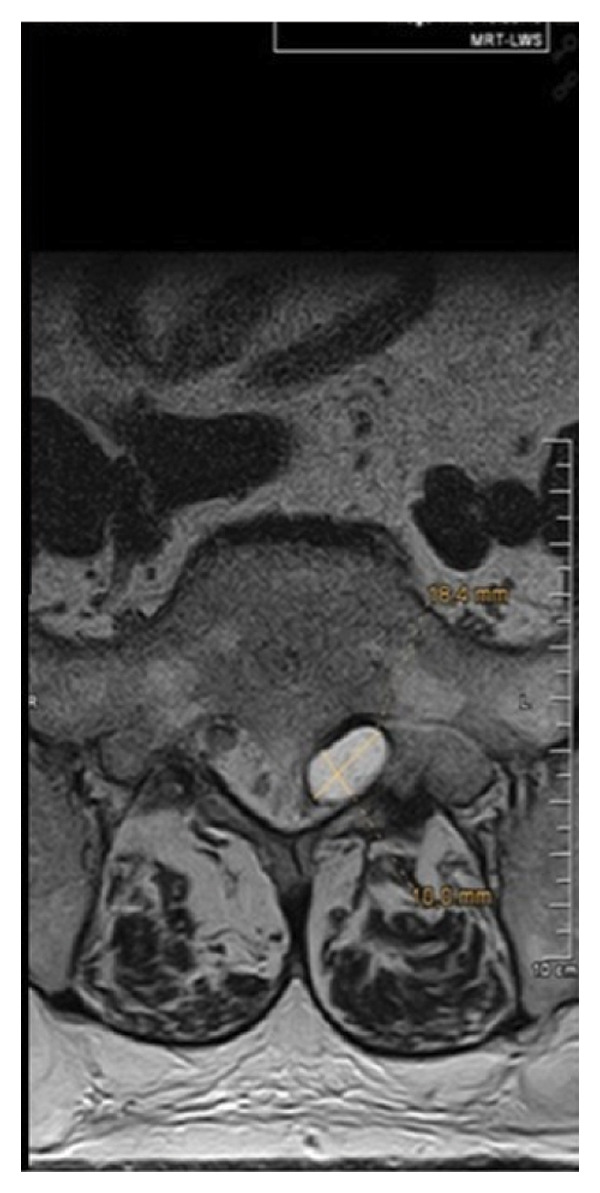
(c)

FIGURE 2Cyst showing similar intensity to cerebrospinal fluid (T2/STIR hyperintense (a and b)/T1 hypointense (c)).

**Figure:**
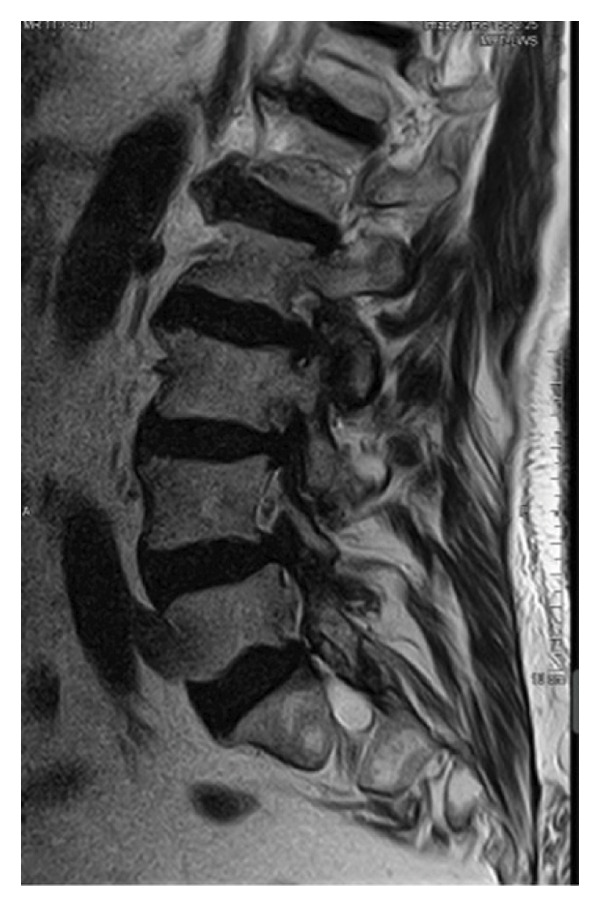
(a)

**Figure:**
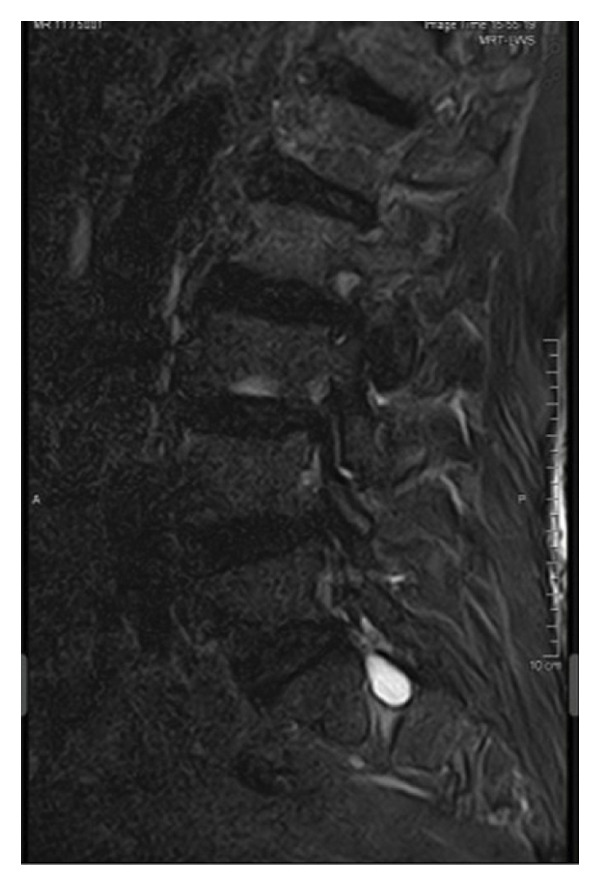
(b)

**Figure:**
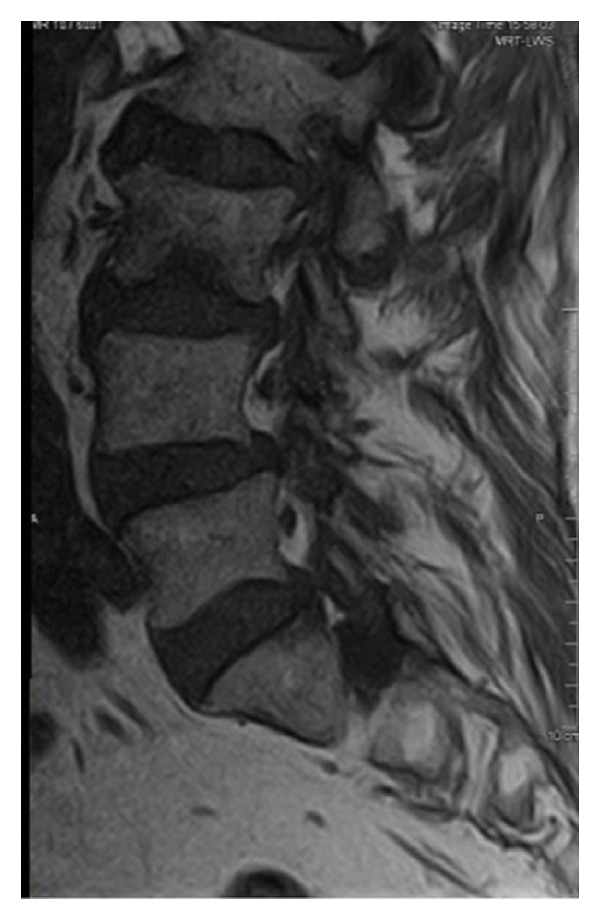
(c)

**FIGURE 4:**
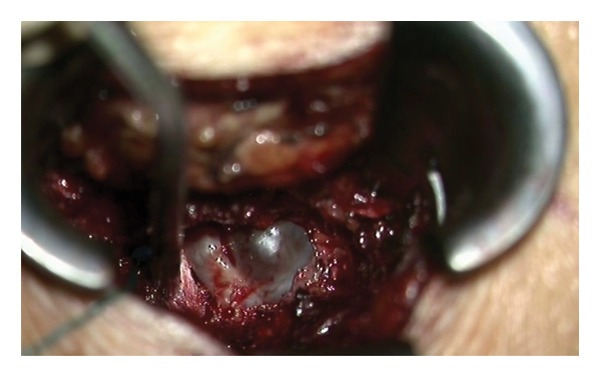
Biloculated cyst revealed under microscope mimicking a Tarlov cyst.

**FIGURE 5:**
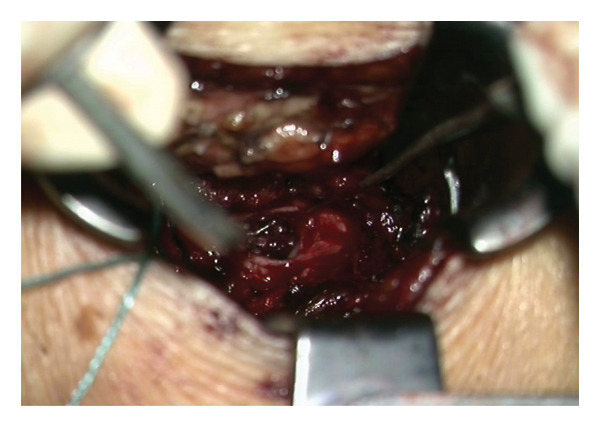
Hemorrhagic content of the cyst.

**FIGURE 6:**
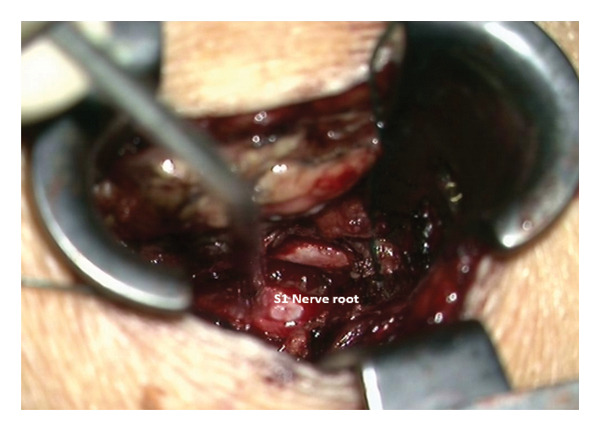
Underlying S1 nerve root after cyst removal.

**FIGURE 7:**
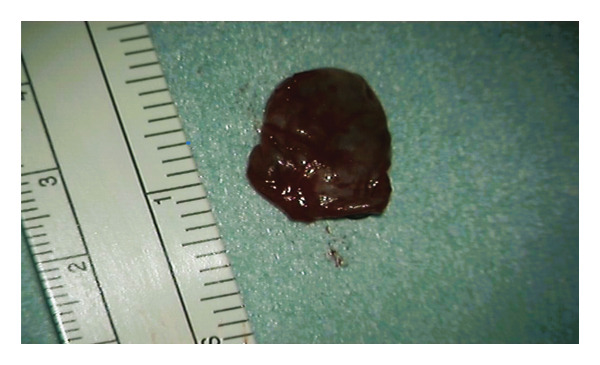
Extracted cyst for histopathological assessment.

The patient was initially treated conservatively with physical therapy, intravenous nonsteroidal anti‐inflammatory drugs, and three C‐arm–guided left S1 nerve root blocks administered over a 1‐week period. Due to insufficient symptom relief, microsurgical intervention was pursued. The patient was placed under general anesthesia and positioned prone. Fluoroscopic guidance was used to identify the correct level at left S1. A standard 3‐cm midline incision was made, and subperiosteal dissection was performed to expose the lamina. A targeted laminotomy was carried out using a 4‐mm diamond burr to carefully remove the laminar bone while preserving surrounding structures. Adequate exposure of the underlying cyst and neural elements was achieved for subsequent inspection and management. Intraoperatively, even under microscopic visualization, the lesion initially appeared consistent with a TC (Figure 4). However, the cyst was found to be filled with hemorrhagic fluid (Figure 5). Careful separation from the underlying S1 nerve root and complete cyst resection were performed. Histopathological examination confirmed the diagnosis of a synovial (facet joint) cyst (Figures 6 and 7). Postoperatively, the patient experienced significant symptom improvement, with marked pain reduction and regression of neurological deficits, though not fully. A repeat MRI performed on postoperative day two showed a full resection of the cyst and resolution of nerve root compression (Figure 8). The patient continued to improve clinically and was advised to continue physical therapy.

FIGURE 8Comparison of preoperative (a) and postoperative (b) T2‐weighted MRI with complete resection of the cyst.

**Figure:**
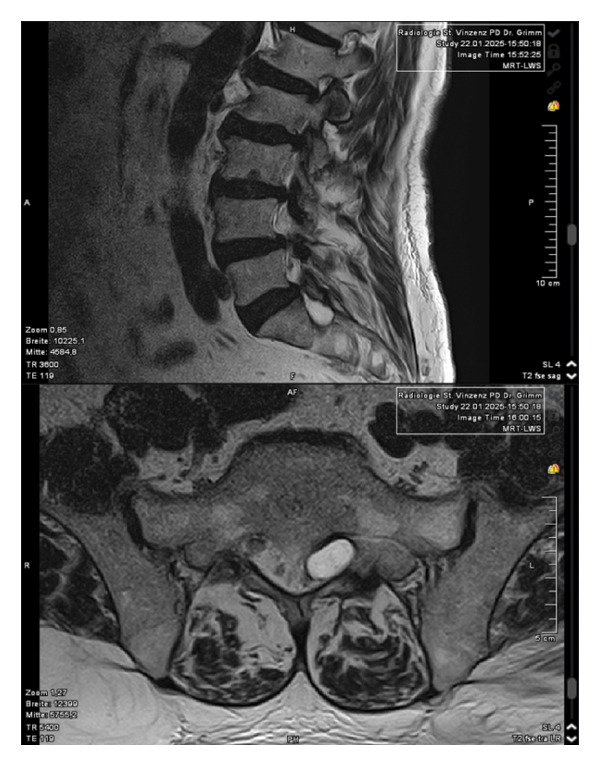
(a)

**Figure:**
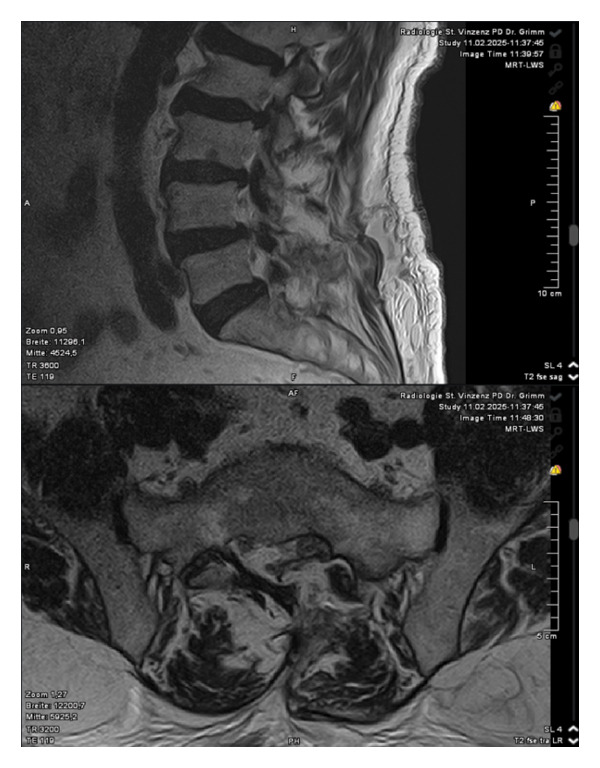
(b)

## 3. Discussion

### 3.1. FJCs and TCs: Diagnostic Challenges

FJCs are most commonly found in the intraspinal compartment of the lumbar spine, most frequently at the L4–L5 level and less commonly at L3–L4 or L5–S1. They are often associated with degenerative changes of the facet joints [7] and have a reported prevalence of approximately 6.5% in the general population. These cysts may cause radicular pain and other neurological symptoms due to the compression of adjacent nerve roots, thereby mimicking conditions such as lumbar disc herniation. TCs, also known as perineural cysts, arise from the spinal nerve root sheath and may occur at any spinal level. They are most frequently reported in the sacral region, but cases involving the lumbar, thoracic, and cervical spine have also been described. They are frequently incidental findings, with a reported prevalence of approximately 4.18%, and are less commonly symptomatic when compared with FJCs, with symptomatic rates reported at approximately 16% for TCs versus up to 54% for FJCs [1–6].

Differentiating between FJCs and TCs can be challenging because of their similar appearance on MRI, as both present as fluid‐filled lesions. However, several radiological features may assist in distinguishing between these entities. FJCs are typically located laterally within the spinal canal and are frequently associated with degenerative changes of the adjacent facet joints. However, facet joint degeneration is common in elderly patients and may also be present in individuals with TCs; therefore, degenerative changes alone cannot reliably distinguish between these entities. In the present case, the inclusion of a FJC in the differential diagnosis was based not solely on degenerative changes at L5–S1 but also on the presence of synovial fluid tracking along the facet joint capsule. Although no definite anatomical communication with the cyst was demonstrated on imaging, these combined findings raised suspicion for a synovial cyst (Figures 1(a), 1(b), and 1(c)). In contrast, TCs arise from the spinal nerve root sheath and are therefore almost always located laterally along the expected course of the nerve root; they are not causally associated with facet joint degeneration, although the two findings may coexist on imaging. In addition, nerve fascicles can often be identified within TCs on preoperative MRI, particularly on T2‐weighted axial images, which may further aid in differentiating them from FJCs. Other spinal meningeal cysts that may be misdiagnosed as TCs on imaging include intrasacral meningoceles, which are typically located centrally within the spinal canal and arise from the distal tip of the sac rather than from a nerve root [8].

A contrast‐enhanced T1‐weighted MRI sequence was not performed, as the lesion demonstrated homogeneous signal characteristics identical to CSF on all noncontrast sequences, without internal septations, solid components, or other features suggestive of a neoplastic process. Based on these imaging findings, the lesion was initially interpreted as a benign cystic entity, and contrast administration was not considered necessary at that stage. Nevertheless, the absence of contrast‐enhanced imaging represents a limitation, as enhancement patterns may further aid in differentiating purely cystic lesions from solid tumors such as schwannomas. In the present case, radiological evaluation did not allow a definitive diagnosis because of the unusual location of the cyst, situated caudal to the S1 pedicle, and the absence of a clear radiological connection to the L5–S1 facet joint. Similar atypical locations of FJCs have been described by Hoover and Pirris [9]. Intraoperatively, the cyst initially resembled a perineural (Tarlov) cyst under microscopic visualization. However, surgical fenestration revealed hemorrhagic rather than clear CSF content. While hemorrhagic content has been reported in TCs, it is typically associated with complications and is not a common finding [10]. In this context, the intraoperative appearance was considered more suggestive of a FJC. Other atypical locations reported in the literature include intraforaminal cysts [11] and cysts migrating into the erector spinae or multifidus muscles [12].

### 3.2. Management Strategies

Both FJCs and TCs may be asymptomatic or symptomatic. Asymptomatic lesions generally do not require surgical intervention unless significant neurological symptoms develop. Conservative treatment—including physical therapy, analgesia, image‐guided injections, and lifestyle modifications—is typically considered first‐line therapy for symptomatic cases [1]. Lutz et al. reported that percutaneous rupture of FJCs may offer a nonsurgical treatment option; however, approximately 40% of patients ultimately required surgery [13]. Similar rates of subsequent surgical intervention (19%–54%) have been reported following failed fluoroscopy‐guided steroid injections [14, 15]. Surgical decompression and excision of the FJC via a targeted laminotomy are therefore indicated in cases of persistent nerve root compression and progressive or functionally limiting neurological symptoms, as was the case in our patient, in whom surgery resulted in immediate and sustained symptom relief. Various surgical approaches have been reported in the literature, including open cyst resection, tubular cyst resection, cyst resection with arthrodesis, endoscopic cyst resection, and percutaneous cyst rupture [16].

For symptomatic TCs, interventional nonsurgical treatment options include CT‐guided cyst aspiration, with or without fibrin glue injection or autologous blood patching [17]. Popular surgical strategies reported in the literature include cyst drainage with wrapping, partial excision with oversewing of the cyst wall (with or without reconstruction of the nerve root sleeve), cyst imbrication, and selective cyst excision while preserving the involved nerve root. Bipolar cauterization for cyst shrinkage, as well as complete resection or clipping of the cyst together with the affected nerve root, is not favored due to the high risk of neurological deficits. Other techniques that are less frequently employed—primarily because of poor long‐term outcomes or high recurrence and complication rates—include cyst fenestration, marsupialization, decompressive laminectomy, lumboperitoneal shunting, and cyst‐to‐subarachnoid shunt placement [18–23].

Although the lesion was ultimately a FJC, the surgical team was prepared intraoperatively for the possibility of a Tarlov (perineural) cyst. The surgeons are experienced in managing both extradural and intradural spinal pathologies through minimally invasive tubular retractors. Had the lesion been a TC, microsurgical dissection under high magnification, careful preservation of nerve root fibers, controlled cyst fenestration or imbrication, and watertight closure with dural repair and sealants would have been performed. Thus, the tubular approach is compatible with safe and effective management of TCs, and contingency plans were in place prior to surgery.

Overall, FJCs and TCs can often be differentiated based on characteristic radiological features; however, atypical location or overlapping imaging characteristics may limit diagnostic certainty. In the present case, although imaging suggested a TC, factors such as the cyst’s caudal migration, hemorrhagic content, and a lack of a clear connection to the facet joint made it unlikely to respond to less invasive interventions such as percutaneous aspiration or fenestration. Surgical management was therefore indicated, allowing direct inspection of the cyst, complete excision, evaluation of cyst contents, and histopathological confirmation, which provided both a definitive diagnosis and effective treatment.

## Funding

No funding was received for this manuscript.

## Conflicts of Interest

The authors declare no conflicts of interest.

## Data Availability

The data supporting the findings of this case report are available from the corresponding author upon reasonable request. All relevant patient information has been deidentified to protect patient privacy.
